# Field Applications of Ultra High Frequency Techniques for Defect Detection in GIS

**DOI:** 10.3390/s18082425

**Published:** 2018-07-26

**Authors:** Jun Xiong, Yuan Wang, Guo-Ming Ma, Qiang Zhang, Shu-Sheng Zheng

**Affiliations:** 1 Guangzhou Power Supply Co., Ltd., Guangzhou 510620, China; xiongj@guangzhou.csg.cn; 2 Key Laboratory of Alternate Electrical Power System with Renewable Energy Sources, North China Electric Power University, Beijing 102206, China; ncepu_ywang@163.com (Y.W.); ncepu_zhangqiang@163.com (Q.Z.); zss4@163.com (S.-S.Z.)

**Keywords:** condition monitoring, partial discharge, sensors, antennas array, location, UHF

## Abstract

The reliable and stable operation of power apparatus is important for the development of GIS. It is important to utilize condition monitoring technologies and anticipate possible failures in advance. Many papers have been published about the partial discharge detection with UHF or X-ray in laboratory, but seldom in field application. Thus, many engineers at project sites are not familiar with the current professional diagnosis techniques. Recently, during the GIS routine data analysis obtained by partial discharge online monitoring system, it was found that the UHF monitoring signals’ developing trend of the 220 kV GIS No. 2 high-voltage side of transformer in phase C at an actual station was abnormal and needed further detection. In order to precisely investigate the problem and then guide the operation and maintenance activities, a series of professional diagnoses were conducted. Three new types of partial discharge detection and positioning methods were applied for accuracy, including UHF partial discharge detection based on multi-stage amplified signal demodulation and multiple weighted averages processing; the partial discharge detection based on the signal radiation hole of insulation disk at the ground connection; and the positioning method based on UHF-SHF. After a series of troubleshooting works, the partial discharge defects have been diagnosed, and the case can be referred in the field monitoring of GIS.

## 1. Introduction

Electrical insulation performance is one of the most significant factors in power systems. Some of the failures in substations are caused by partial discharge (PD) phenomena suffered by the electric equipment. The possible causes of PD include the insulation damage caused by overvoltage or impulse voltage, metal particles produced during the installation, and natural aging damage [1,2,3,4]. Although PD may be slight at the beginning, some failures would happen among the high voltage apparatus with its development [5,6,7]. Therefore, it is essential to utilize condition monitoring technologies in the power system to achieve the timely diagnosis of PD and allow early responses.

Therefore, many PD defect detection methods have been studied, such as, pulse current detection, acoustic detection, ultra-high frequency (UHF) detection, and X-ray inspection [8,9]. Many electrical phenomena will be accompanied with the occurrence of PD, for instance, electrical current pulses and electromagnetic radiation. The frequency of electromagnetic waves extended well into the UHF range (300 MHz~3 GHz) [8,9,10,11,12]. Thus, the UHF monitoring technique is adopted into the PD detection [12,13,14,15,16,17]. It has the advantages of high frequency of detection signal, less interference from outside, and easy to install. It is suitable for live tests in actual operation. Typical UHF methods are mainly based on the UHF signals such as time of arrival, time difference of arrival and angle of arrival, among them, the time difference of arrival is widely used in PD positioning [13,14]. However, as the distance from the PD source increases, the signals will be greatly attenuated, which makes it difficult to determine the starting pulse time of the signals, and results in a large error [18,19]. In order to achieve the goal of high sensitivity, the UHF antenna sensors used to collect the PD signals are key research objects in UHF detection technology. Some researchers use multiple or new designed antennas to receive UHF signals, for example, L-shaped, disc-type antenna [20,21,22,23,24,25,26]. Also, X-ray inspection is a non-destructive testing method, which uses X-rays to penetrate materials and find out defects through the attenuation characteristics in the material [27,28]. The wavelength of the X-ray is very short, generally range from 0.001 to 0.1 nm. X-rays propagate linearly at the speed of light and are not affected by electric and magnetic fields. These features make it is suitable for industry application. 

However, the methods mentioned above are used rarely in practical operation, so it is necessary to utilize them in field applications. What’s more, six short circuit faults occurred in an area of south China. Five of them were insulation faults and one was a current-carrying fault. The faults that happened among equipment were still not effectively controlled. There were four fault stations equipped with online monitoring devices, but none of them could effectively find the partial discharge before the fault. Insulation faults are common and will occur several times a year. Therefore, the effectiveness of online monitoring devices needs to be further improved.

In August 2015, the development trend of the UHF monitoring signals in the 220 kV GIS No. 2 high-voltage side of transformer in phase C were abnormal, the amplitude of the discharge reached 900–1000 mV and the alarm frequency increased. From a historical perspective, the daily average number of alarms has increased from 600 s in August 2015 to about 900 in November 2015, which needed urgent attention. In order to find out the problem exactly and then guide the operation and maintenance, a series of professional diagnoses were conducted, which included: (1) UHF partial discharge detection technology based on multi-stage amplified signal demodulation and multiple weighted average processing. This was used to obtain the average waveform, which improved the signal-to-noise ratio and improved the accuracy. Then the position was determined by the time differences between different measuring points. (2) The partial discharge detection based on the signal radiated from the hole of insulation disk at the ground connection was applied for positioning by the time difference. (3) The positioning method based on UHF-SHF was applied. Four UHF-SHF sensors were arranged along the circumference of the spacer at equal distances. If a partial discharge occurs from the coaxial direction of the conductor, the time difference between the antenna array will be close to 0. If not, when the PD source deviates away from the coaxial direction (or PD source was next to one of the antennas), the time difference between the four antenna arrays would increase. With the help of the advanced online condition monitoring technologies, suspected partial discharge phenomena were detected, which effectively narrowed the scope of inspections and provided specific guidance in a timely way. 

## 2. Experimental Arrangement

### 2.1. Experiment Setup

The 220 kV GIS operating in the substation was manufactured in May 1998. The circuit breakers’ rated current is 2 kA, rated short-circuit breaking current is 40 kA. They work using a pneumatic operating mechanism. The UHF partial discharge online monitoring system was installed on the GIS in 2010.

**Figure 1:**
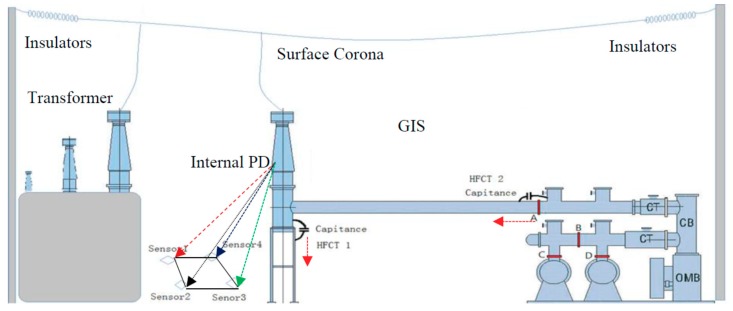
The diagram of 220 kV GIS high-voltage interval of phase C.

The 220 kV GIS adopts sectionalized double-bus configuration with a total of 12 intervals (five line intervals, all of which are cable outlets; three overhead intervals, all of which are overhead line outlets). Also, the line intervals and high-voltage side of transformer are divided into five to six gas chambers. There are three gas chambers in the bus-bar and segmental intervals. The bus-bar intervals are divided into three gas chambers. There are no cone-type spacers in each bus-bar, which means only one gas chamber for each bus-bar. The configuration of the 220 kV GIS high-voltage interval is shown in Figure 1.

### 2.2. Experiment Method

**Figure 2:**
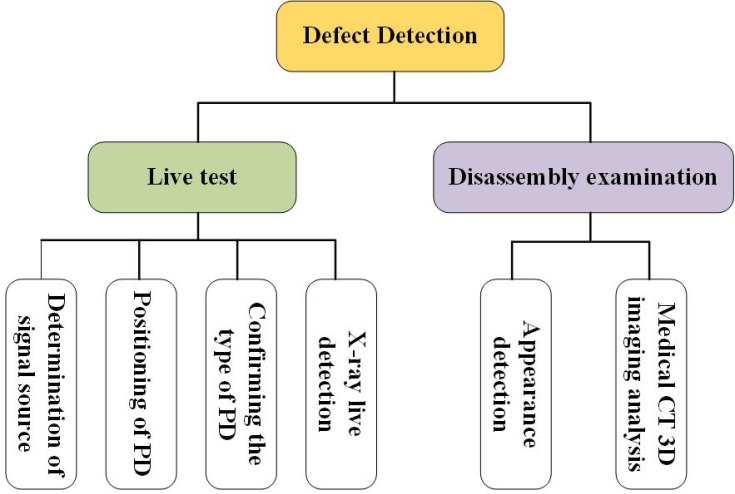
The detail partial discharge detection methods.

In order to accurately check out the cause of abnormal signals, live tests and disassembly examinations were carried out, respectively. The GIS partial discharge live test was divided into four steps and got carried out in turn. In the first step, the UHF partial discharge detection technologies from different manufacturers were used to verify the possible fault positions based on the online monitoring signals. In the second step, the technique of wraparound method (four UHF-SHF sensors in the system were arranged along the circumference of the spacer at equal distances, and then faults were positioned through the time difference between signals received by four sensors) based on UHF partial discharge detection; or SHF partial discharge detection technique (RF antenna) was adopted to analyze the signal source analysis. The detail detection methods are shown in Figure 2. 

We could preliminarily determine whether the signals were generated by the spatial signal interference, the conduction by the transformer, or originated from the inside of the GIS device. In the third step, time difference method based on UHF was used to localize the partial discharge source. In the fourth step, considering the internal components and the type of discharge, X-ray detection was used to inspect the hidden features of suspicious fault locations, and observe the characteristics changes simultaneously. Then the disassembly examination was divided into two parts: appearance detection and medical 3D imaging 

## 3. Results and Discussion

### 3.1. Live Test

#### 3.1.1. Determination of Signal Source

Live test means the detection is conducted when a suspicious fault or fault occurs in the substation which is in operation. Also, live tests should be conducted within a certain period of time to find out possible failures and then ensure the reliable operation of the equipment. UHF live test technology based on DMS, which frequency ranges from 300 MHz to 1.5 GHz, was used in the test. There were totally 12 test points for this interval (four cone-type spacers named as A, B, C, D were available as measuring points in each phase), which is shown in Figure 1.

**Figure 3:**
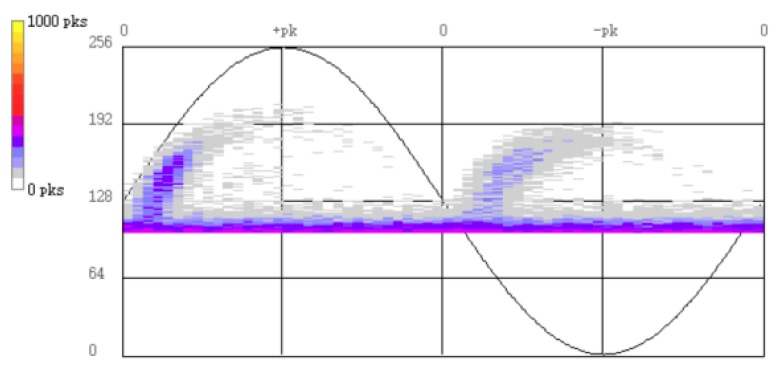
DMS UHF live test PRPD spectrum of measuring point A.

**Figure 4:**
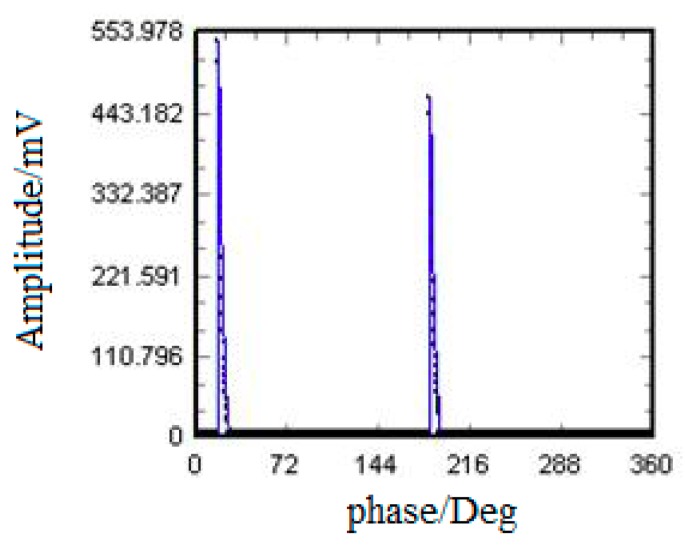
PRPD figure of online monitoring at measuring point A.

The live test results are reported in the Figure 3. It is found that among the 12 measuring points, suspected signals were only detected at measuring point A, phase C. And no suspected signal was observed at the other 11 measuring points. Figure 4 shows the phase resolved partial discharge (PRPD) spectrum of the measuring point A by online monitoring (online monitoring is under working condition all the time, no matter whether there are any possible faults). From Figure 3 and Figure 4, it is obvious that the phase characteristics obtained by the live test were consistent with the results of online monitoring. That is to say, the abnormal signals at measuring point A of phase C needed more attentions and should have to distinguish the source of the signals. Also, this proved the reliability of the detection method.

Since obvious signals were only detected at measuring point A, and similar signals were not measured at other measuring points, it can be preliminarily judged that the signal was less likely to be transmitted from the bus-bar. Then further analysis was needed to determine whether the signal obtained at the measuring point A was spread from the direction of the transformer or from the space around the measuring point A. Thus, one sensor measured away from the point to be tested gradually to figure out whether signal was produced by an internal source or a spatial source.

**Figure 5:**
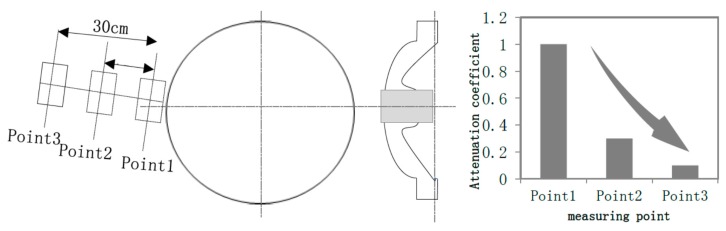
Sensor position for detection and the attenuation characteristics.

**Figure 6:**
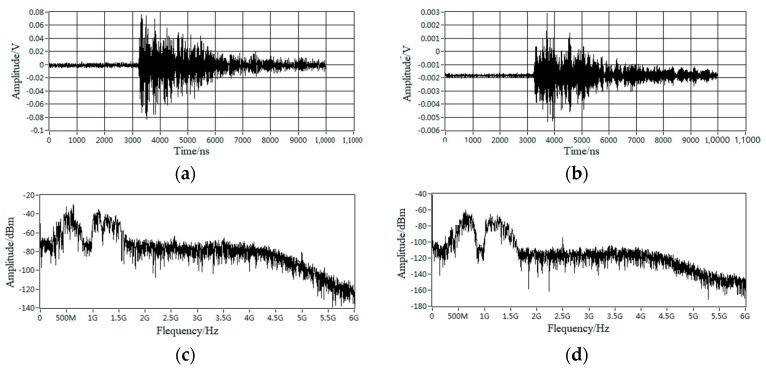
Signal characteristics at point 1 and point 3. (**a**) Time domain signal of point 1; (**b**) Time domain signal of point 3; (**c**) Frequency spectrum of point 1; (**d**) Frequency spectrum of point 3.

Firstly, placing the DMS UHF sensor at Point 1, Point 2, and Point 3 in turn to record the signal’s amplitude, and defining the signal’s amplitude at Point 1 as 1, as shown in the left figure of Figure 5. According to the measuring results, the phase characteristics of the signals measured at the above three positions were similar, but the amplitudes were decreased in order. Compared to Point 1, the signal’s amplitude of Point 2 and Point 3 were only 30% and 10% respectively, as shown in Figure 5 and Figure 6. The amplitude of the signal decreased when the distance between sensor and the spacer got increased. In addition, when the antenna’s receiving direction was back to the spacer, the pre-detected signals would disappear immediately. The above results all indicated that the signals obtained at measuring point A were generated by an internal source.

Secondly, it was necessary to further confirm the signals obtained at measuring point A whether occurred inside the GIS or transmitted from the direction of the transformer. Three methods were adopted. First, the use of infrared and ultrasonic to detect the running risks on both sides of the bushing. Second, between transformer and the bushing of GIS, UHF-SHF partial discharge detecting and positioning antenna arrays were arranged to detect the hidden danger of discharge. Third, the coupling capacitances were arranged at the base of bushing (phase C) and measuring point A, then the signals’ characteristics of the two positions detected by HFCT were used to further determine whether it was caused by the signal interference transmitted from transformer. The UHF-SHF sensors and HFCT sensors’ arrangements are shown in Figure 1. By this method, it can be proved that the internal source detected at measuring point A was not transmitted from the transformer but likely occurred within the GIS.

In addition, in order to confirm the above conclusion, the high frequency pulse current method was adopted. Two high-frequency coupling capacitors were arranged in the positions of the bushing and the spacer shown in Figure 1, and each coupling capacitor circuits had one HFCT sensor applied to. The amplitude of the signals obtained by two HFCT sensors were used to judge the signals whether originated from the GIS bushing or other locations within the GIS chamber.

We take equivalent time length and equivalent frequency to separate the discharge pulse waveform, which means multiple discharge sources got separated. It reflects the number of PD sources in the field [29]. 

**Figure 7:**
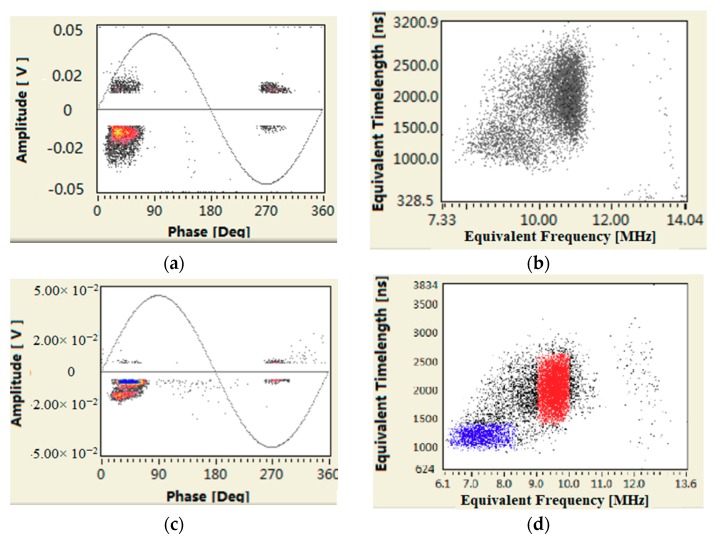

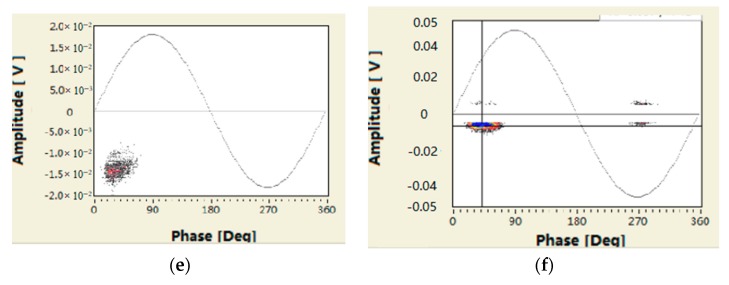
Results obtained by pulse current method. (**a**) HFCT 2 PRPD spectrum; (**b**) HFCT 2 Equivalent time-frequency spectrum; (**c**) HFCT 1 PRPD spectrum; (**d**) HFCT 1 Equivalent time-frequency spectrum; (**e**) HFCT 1 PRPD spectrum blue cluster; (**f**) HFCT 1 PRPD spectrum red cluster.

**Figure 8:**
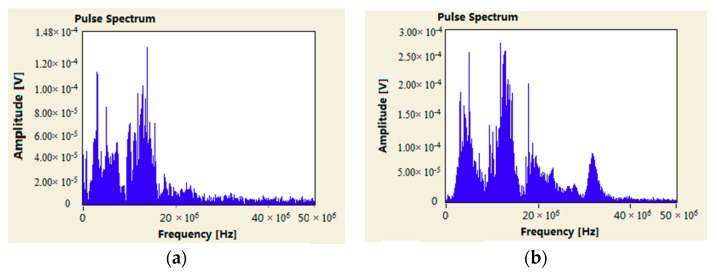
Pulse frequency waveform characteristics of two positions of HFCT1 and HFCT2. (**a**) HFCT 1; (**b**) HFCT 2.

According to the data shown in Figure 7, for HFCT 2 (Figure 7a,b) installed at the cone-type spacer, the PRPD spectrum had a phase characteristic of 180°, the single pulse amplitude could up to 180 mV, and the main frequency was as high as 20 MHz to 35 MHz. It was identified as a 100% internal discharge signal. For HFCT 1 (Figure 7c,d) installed on the GIS bushing base, the obtained signals could be divided into two types, presented in red and blue. The PRPD spectrum corresponding to the blue one was identified as an external corona signal. The PRPD spectrum corresponding to the red one had a phase characteristic of 180°, the single pulse amplitude can up to 85 mV, the dominant frequency ranged from 20 MHz to 23 MHz, it was identified as an internal discharge signal. Figure 8 shows that both PRPD spectrum and single pulsed current waveforms had some similarities, and the signals received by HFCT 2 were stronger than HFCT 1. What’s more, with the use of the Ultra 9000 Ultrasonic Tester, no abnormality was detected in the high-voltage side of transformer. The humidity and component were tested at bus-bar branch bushings, disconnector, and circuit breakers. No abnormal results were found. The results further indicated that the internal source obtained at the spacer (measurement point A) was not transmitted from the transformer, but it was likely happening inside the GIS.

#### 3.1.2. Positioning of PD

The conclusion obtained was based on the traditional portable UHF detecting method. Signals only got detected at measuring point A, and no signals are measured at other points. According to the principle of the time difference method, signals only obtained at one point are not enough to determine the fault position. It means the scope of inspection would have to cover all equipment of phase C, which is not desirable. In order to narrow the inspection scope, three new types of partial discharge detecting and positioning methods were applied: (1) UHF partial discharge detection technology based on multi-stage amplified signal demodulation and multiple weighted average processing. (2) The partial discharge detection based on the signal radiated from the hole of insulation disk at the ground connection. (3) The positioning method based on UHF-SHF.

- UHF partial discharge detection based on multi-stage amplified signal conditioning and multiple weighted averages

**Figure 9:**
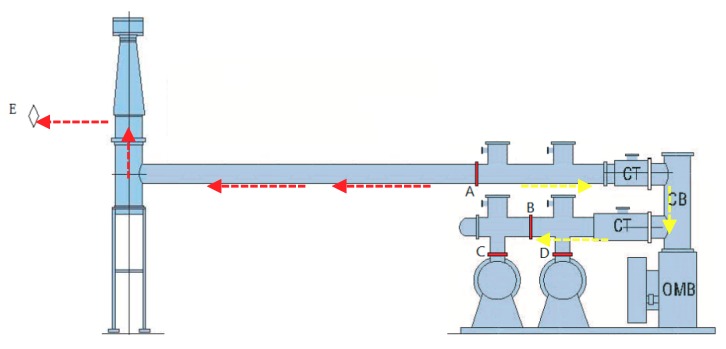
Schematic of time difference positioning method.

As shown in Figure 9, since the UHF signal amplitudes at B and E were generally small, 1000 acquisitions were used to obtain the average waveform to improve the signal-to-noise ratio. However, multiple averaging method would cause the weakening of the wave front, which would lead to large fluctuations. In addition, due to the minimum range of the oscilloscope is 5 mV/division, the accuracy of the acquisition was also difficult to be guaranteed when measuring the tiny signals at B and E. Therefore, five waveform data were collected for each measuring point, and the average value was used to minimize the influence of the above factors.

The DMS UHF sensor and the UHF amplifier were used to measure signals at points A and B simultaneously, and the position was judged by the time difference. Point B was a cone-type spacer used to isolate the gas chamber between two busbars. The DMS sensor was attached to the cone-type spacer where it had no shielding. Also, the DMS signal at point E was measured. Details of the data are recorded in Table 1.

Taking the speed of signal propagated in the GIS as the speed of light, the average distance between the point A and B was 6.17 ± 0.6 m. The actual measured distance between point A and B was 6.14 m. And the average distance between the point A and E was 7.47 ± 1.2 m. The actual measured distance between point A and E was 7.14 m.

To sum up, the internal partial discharge position may be within 1 m around the cone-type spacer, which was adjacent to the grounding switchgear. The scope covers the switchgear, grounding switchgear and some of the bus-bars.

- The partial discharge detection based on the signal radiated from the hole of insulation disk at the ground connection

**Figure 10:**
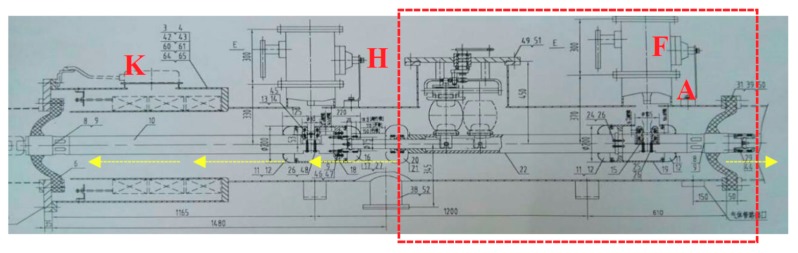
The sensors layout which improved the precise positioning.

**Figure 11:**
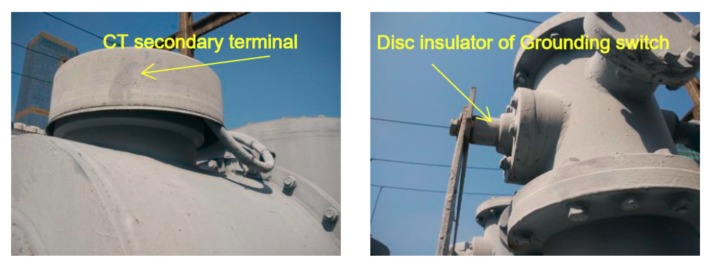
Appearance of CT secondary terminal and disc insulator of grounding switchgear.

In order to minimize the inspection scope, a time-difference locating method based on signal radiated from the hole of insulation disk at the ground connection was applied. According to the GIS structure, the grounding switchgear and grounding rod on both sides of disconnector were led to the ground by the external ground connection, and epoxy spacer were used to insulate with the chamber. Therefore, the UHF signals might radiate from this location and get detected by the UHF external sensor, point F, as shown in Figure 10, and the position showed in Figure 11.

The DMS UHF sensor and the UHF amplifier were used to measure points A and point F simultaneously, and the position was judged by the time difference. The signal collected at point F can be distinguished from the background signal after averaging 839 times. The V_pp_ was about 25 mV. From the results of spectrum analysis, the energy was about −10 dB smaller than the results at the cone-type spacer. From 5 series of sample data, the average time difference was −0.7 ns. Therefore, the distance between point A and point F was about 57 cm. To sum up, the signal may occur between the disconnector and the grounding switchgear.

- The positioning method based on UHF-SHF

**Figure 12:**
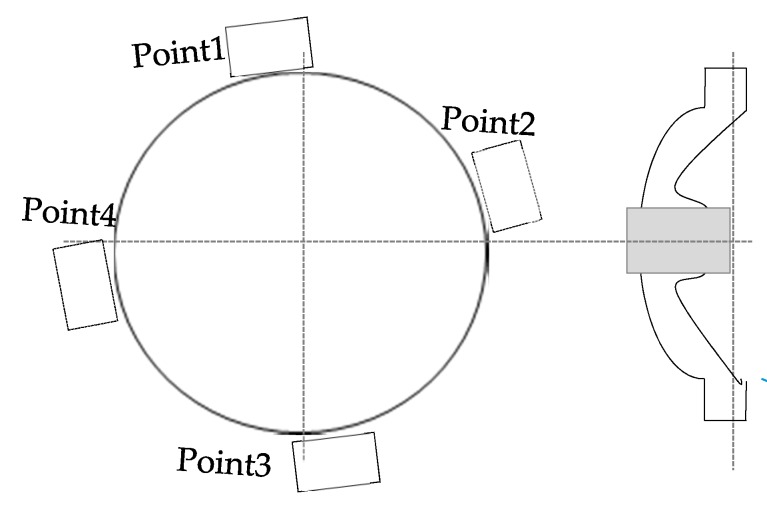
Distribution diagram of antennas array.

The principles of UHF-SHF based detection and location technology are as below: four UHF-SHF sensors in the system are arranged along the circumference of the spacer at equal distances. If the partial discharge occurs from the coaxial direction of the conductor, the time difference of the antenna array will be close to 0. If not, when the PD source deviates away from the coaxial direction (or PD source is next to one of the antennas), the time difference between the four antenna arrays will get increased. The layout of the four antennas array is shown in Figure 12, and the time difference of four points are shown in Table 2.

Results show that Point 1 firstly detected the signal and Point 4 detected the signal almost simultaneously with Point 1, then Point 2, and Point 3. However, the maximum time difference between the four antenna arrays did not exceed 0.3 ns. In summary, it can be known that the discharge source was closer to the cone-type spacer or the non-axis position which was far away from the cone-type spacer. The scope covers transmission spacer, grounding switchgear and bus branch supporting spacer of phase C.

#### 3.1.3. Confirming the Type of PD

**Figure 13:**
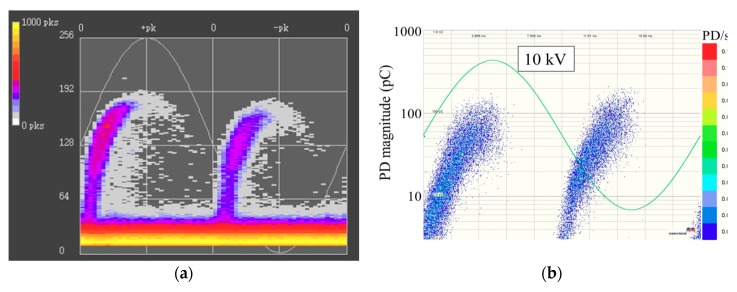
Comparison of measured spectrum with typical spectrum. (**a**) The measured spectrum; (**b**) the typical PRPD pattern when there are air bubbles inside the solid epoxy.

In order to find out hidden dangers and provide reference for subsequent defect analysis, it is necessary to perform discharge type identification on the partial discharge signals collected in the field. The identification results of UHF online monitoring showed that the probability of void was 45%, the possibility of floating potential was 35%, and the probability of free metal particle discharge was 20%. Comparing the measured spectrum with the typical defect pattern, the results are shown in Figure 13. It shows that the measured spectrum was close to the PRPD spectrum when there were air bubbles inside the solid epoxy material.

#### 3.1.4. X-ray Live Detection

**Figure 14:**
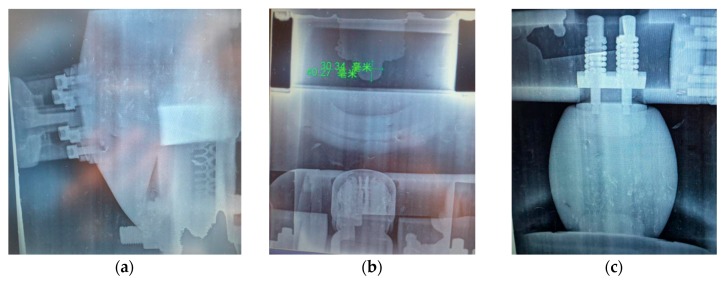
Typical X-ray flaw detection diagram of phase C. (**a**) cone-type spacer; (**b**) grounding switchgear; (**c**) supporting spacer.

X-ray detection was performed on the possible partial discharge locations mentioned in Section 3.1.2. No obvious abnormalities were found. The X-ray detection spectrum are shown in Figure 14.

The latest experimental research experience shows that when X-rays are applied to a simulated discharge defect within a solid material, the number of discharge pulses per unit time increases, and the discharge interval in the PRDP spectrum gets broadened. For this reason, when X-ray inspections were performed on suspicious positions inside the GIS, the partial discharge characteristics of the measuring points A were observed simultaneously.

To summarize, according to the conclusions gained by live tests, the scope needs to conduct the disassembly examination was the disconnector and the bus-bar branch chamber of phase C. The parts needed especially inspection mainly include: (1) grounding switchgear; (2) transmission spacer of disconnector; (3) Support spacer region on the side of the busbar branch. Possible types of PD defects were air gaps or void inside the solid insulating material.

### 3.2. Disassembly Examination

**Figure 15:**
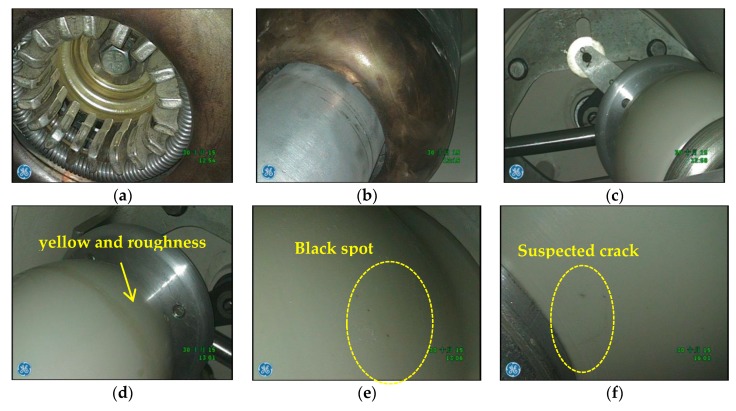
Appearance detection results obtained by endoscope. (**a**) static contact; (**b**) Appearance of static contact; (**c**) transmission spacer; (**d**) Synthetic seam on transmission spacer; (**e**) Surface of transmission spacer; (**f**) Surface of cone-type spacer.

After the cover of phase C get opened, the appearance of the transmission spacers, static contacts, inner wall of chamber, and the cone-type spacers adjacent to the grounding switchgear, as well as the supporting spacers and the inner wall of the bus-bar were checked by endoscope. The results are shown in Figure 15.

The transmission part of disconnector, conductor of grounding switchgear and static contact were inspected. There were no discharge traces among them. The main problems found in the appearance inspection include: (1) 2 to 3 cm suspected cracks on the cone-type spacer; (2) yellowing and local unevenness in the position of the transmission spacer; (3) black spots on the surfaces of cone-type spacers, transmission spacers, and supporting spacers. The above phenomena were consistent with the problems found by the endoscope. All problematic spacers were replaced at once. 

In order to fully inspect whether there were other hidden dangers within the replaced spacers, medical CT 3D imaging analysis was performed on them. With 3D reconstruction technology, 3D slicing analysis can be performed on the spacer to fully grasp the internal state of the spacers.

**Figure 16:**
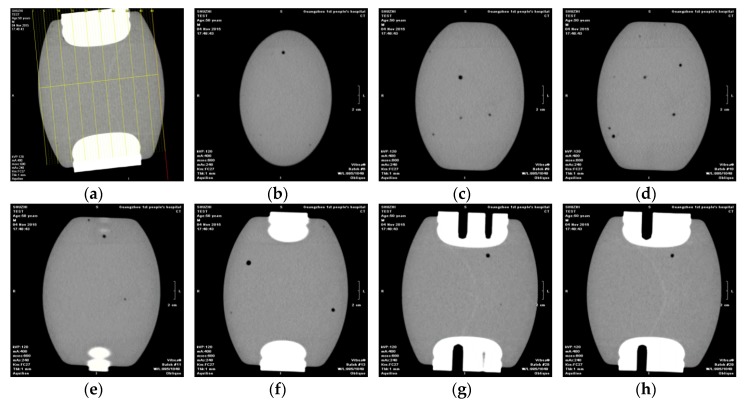
Inspection results of transmission spacers of disconnector. (**a**) Slice diagram; (**b**) 6# slice; (**c**) 9# slice; (**d**) 10# slice; (**e**) 11# slice; (**f**) 13# slice; (**g**) 28# slice; (**h**) 29# slice.

The transmission spacer was cut into 44 slices which sizes were about 1.5 mm. It was mainly found that about 1/3 of the slices contained air bubbles of different sizes (because the density of that area was −600 HU to −900 HU. The density of air was −1000 HU, the density of bone was −1 HU). Typical inspection results of transmission spacers of disconnector are shown in Figure 16.

**Figure 17:**
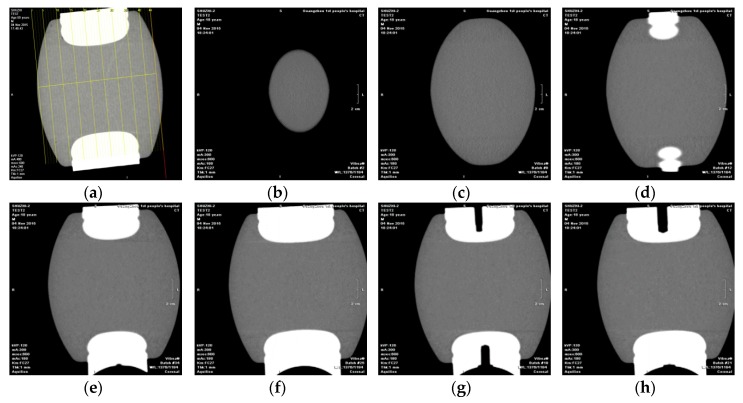
Inspection results of supporting spacers in busbar branch chamber. (**a**) Slice diagram; (**b**) 6# slice; (**c**) 9# slice; (**d**) 10# slice; (**e**) 11# slice; (**f**) 13# slice; (**g**) 28# slice; (**h**) 29# slice.

For the bus-bar supporting chamber supporting spacers, they were cut into 50 slices which sizes were about 1.2 mm, and no hidden troubles such as internal air bubbles and gas gaps were found. Typical inspection results of supporting spacers in busbar branch chamber results are shown in Figure 17.

Through the medical 3D imaging technology it was found that: (1) 2~3 cm cracks appeared on the surface of cone-type spacers, and did not clearly penetrate into the interior of the epoxy material, and the cause of their formation need to be further analyzed. No faults such as air bubbles were found inside the supporting spacers; (2) there were multiple air bubbles inside the transmission spacer of the disconnector.

## 4. Discussion and Conclusions

A variety of field test methods used in the actual operating conditions fully demonstrated the correctness of the online condition monitoring and live tests in finding out suspected partial discharge through phase characteristics. Also, HFCT sensors were used to clarify the numbers of PD types and roughly clarified the PD source were inside the GIS.

Three types of partial discharge detecting and positioning methods were applied successfully to narrow the scope of inspections gradually: (1) UHF partial discharge detection based on multi-stage amplified signal demodulation and multiple weighted averages processing. It confirmed that the internal PD position may be within 1 m around the cone-type spacer adjacent to the grounding switchgear. The scope covered the switchgear, grounding switchgear and some of the bus-bars. (2) The partial discharge detection based on the signal radiated from hole of insulation disk at the ground connection, which found out that the signal may occur between the disconnector and the grounding switchgear. (3) The positioning method based on UHF-SHF, sensors were arranged along the circumference of the spacer at equal distances. It can be known that the discharge source was closer to the cone-type spacer or the non-axis position which was far away from the cone-type spacer. The scope covered transmission spacer, grounding switchgear and bus branch supporting spacer of phase C.

X-ray methods and typical PRPD spectra were used to figure the type of PD. It found out that possible types of PD defects were air gaps or voids inside the solid insulating material. After live tests, partial discharge defects in GIS were diagnosed and confirmed by disassembly examination. The fault types and location where the fault occurred were further confirmed.

In this case, the combination of identification technology of partial discharge including UHF, SHF, HF, X-ray were used, the method and processes can be referred to in the field monitoring of GIS. Also, the results were compared with the theoretical research results obtained in laboratory, which enhanced the confidence of accurately determining hidden dangers.

After the maintenance, the power had been delivered properly. UHF online monitoring and on-site live tests did not find any partial discharge characteristic signals, and the obtained signals were mainly white noise interference signals. This showed that the hidden dangers have been eliminated.

In further research, the effect of X-rays on detecting partial discharge characteristics of GIS should be studied. The mechanism for how X-rays stimulate the internal discharge intensity of GIS can be studied with a GIS scale model, and the research on the blocking effect on the partial discharge signal propagation should be done. The relevant research conclusions will guide for the future diagnosis of similar problems.

## Figures and Tables

**Table 1:** Time difference between point B/E and point A.

	Group 1	Group 2	Group 3	Group 4	Group 5	Vpp	Energy Loss	Time Differences	Distance
Point B	20.20 ns	19.65 ns	19.35 ns	22.50 ns	21.10 ns	≈3 mV	–30 dB	20.56 ns	6.17 m
Point E	21.00 ns	26.35 ns	22.95 ns	28.80 ns	25.35 ns	≈2 mV	–35 dB	24.89 ns	7.47 m

**Table 2:** Time difference of detected signals between four points.

	Point 1	Point 2	Point 3	Point 4
Group 1	0 ns	0.20 ns	0.29 ns	0.05 ns
Group 2	0 ns	0.17 ns	0.30 ns	0.00 ns
Group 3	0 ns	0.12 ns	0.27 ns	0.10 ns
Group 4	0 ns	0.15 ns	0.29 ns	0.06 ns
Group 5	0 ns	0.13 ns	0.26 ns	–0.03 ns
Average time difference	0 ns	0.154 ns	0.282 ns	0.036 ns
